# Economic Evaluation of Interventions for Prevention of Hospital Acquired Infections: A Systematic Review

**DOI:** 10.1371/journal.pone.0146381

**Published:** 2016-01-05

**Authors:** Habibollah Arefian, Monique Vogel, Anja Kwetkat, Michael Hartmann

**Affiliations:** 1 Center for Sepsis Control and Care (CSCC), Jena University Hospital, Jena, Germany; 2 Hospital Pharmacy, Jena University Hospital, Jena, Germany; 3 Center for Clinical Studies, Jena University Hospital, Jena, Germany; 4 Department of Geriatric Medicine, Jena University Hospital, Jena, Germany; Leibniz Institute for Prevention Research and Epidemiology (BIPS), GERMANY

## Abstract

**Objective:**

This systematic review sought to assess the costs and benefits of interventions preventing hospital-acquired infections and to evaluate methodological and reporting quality.

**Methods:**

We systematically searched Medline via PubMed and the National Health Service Economic Evaluation Database from 2009 to 2014. We included quasi-experimental and randomized trails published in English or German evaluating the economic impact of interventions preventing the four most frequent hospital-acquired infections (urinary tract infections, surgical wound infections, pneumonia, and primary bloodstream infections). Characteristics and results of the included articles were extracted using a standardized data collection form. Study and reporting quality were evaluated using SIGN and CHEERS checklists. All costs were adjusted to 2013 US$. Savings-to-cost ratios and difference values with interquartile ranges (IQRs) per month were calculated, and the effects of study characteristics on the cost-benefit results were analyzed.

**Results:**

Our search returned 2067 articles, of which 27 met the inclusion criteria. The median savings-to-cost ratio across all studies reporting both costs and savings values was US $7.0 (IQR 4.2–30.9), and the median net global saving was US $13,179 (IQR 5,106–65,850) per month. The studies’ reporting quality was low. Only 14 articles reported more than half of CHEERS items appropriately. Similarly, an assessment of methodological quality found that only four studies (14.8%) were considered high quality.

**Conclusions:**

Prevention programs for hospital acquired infections have very positive cost-benefit ratios. Improved reporting quality in health economics publications is required.

## Introduction

Hospital acquired infections (HAIs), also called nosocomial infections, are a serious public health problem and a major cause of morbidity and mortality[1]. Moreover, HAIs can prolong the length of hospital stays and increase costs for healthcare systems [2]. The annual financial losses due to HAIs, including direct costs only, are estimated at approximately € 7 billion in Europe and US $6.5 billion in the USA [3,4]. In 2002, approximately 1.7 million infections (4.5 per 100 admissions) were acquired in US hospitals [5]. The most frequent HAIs are urinary tract infections, surgical wound infections, ventilator-associated pneumonia, and primary bloodstream infections [1]. A recent meta-analysis of HAIs showed that central line–associated bloodstream infections are tied up with the highest costs ($45,814), followed by ventilator-associated pneumonia ($40,144) and surgical wound infections ($20,785) [6]. Several systematic reviews have inspected the clinical effect of interventions for HAI prevention [7–9]. Most of the studies included in these reviews showed a reduction in the number of HAIs. However, the economic benefit of such interventions is not clear.

Therefore, we conducted this systematic review to provide a cost-benefit estimation for HAI prevention and to examine the quality of economic studies and their reporting.

## Methods

### Data Sources and Search Strategy

Our systematic review conforms to recommendations in the Preferred Reporting Items for Systematic Reviews and Meta-Analyses (PRISMA) statement (S1 Table) [10] and guidance from the Campbell and Cochrane Economics Methods Group on incorporating economic evidence into systematic reviews [11].

Searches of eligible studies were conducted in Medline via PubMed and the National Health Service Economic Evaluation Database (NHS EED) to identify relevant articles in English and German published in a five year period between January 2009 and January 2014 with an abstract available for review.

We used several search terms, and keywords were matched to database-specific indexing terms (Mesh and ti). We used the operator AND to link keywords with different meanings and the operator OR for keywords with similar meanings. S2 Table shows our search strategy for eligible publications in PubMed.

### Selection Criteria

All studies found were reviewed for eligibility by applying the PICO (patient problem or population, intervention, comparison, and outcomes) question format. Publications identified in the search of the two databases were combined and duplicates were removed.

#### Population

We included studies assessing interventions intended to prevent the four most common HAIs (urinary tract infections, surgical wound infections, hospital-acquired pneumonia, and primary bloodstream infections). There was no age or gender restriction in this systematic review.

#### Types of interventions

The interventions of interest were measures reducing person-to-person transmission (hand decontamination, personal hygiene, clothing, masks, gloves and safe injections); measures preventing transmission from the environment (cleaning of the hospital environment, use of hot/superheated water, discussion of patient equipment); and measures proven effective for the prevention of urinary tract infections, surgical site infections (SSI), pneumonia and vascular device infections that used the World Health Organization (WHO) guidelines for HAI prevention [1]. Studies involving only an evaluation of any other preventive measures were excluded.

#### Control/Design

We included quasi-experimental and randomized trails, while articles without an explicitly formulated study design or method were excluded. We also excluded cross-sectional studies, reviews, guidelines, studies of pure mathematics, studies published as an abstract only and studies using a simulation or modeling published data.

#### Outcome measures

In this review, cost-effectiveness analyses (CEAs), cost-benefit analyses (CBAs), cost-minimization analyses (CMAs) and cost-utility analyses (CUAs) were included. Studies lacking quantitative economic parameters or reported outcomes were excluded.

### Data Extraction

Two reviewers (H.A., M.V.) independently applied inclusion and exclusion criteria and extracted the data from eligible studies by screening titles, abstracts and full-text articles. Differences were resolved by discussion with a third review author. The reviewers documented the reasons for excluding articles from the review.

We extracted the characteristics and results of included health economics studies using a standardized data collection form [11]. We also consulted previous systematic reviews of health economics studies to improve our data collection form [12,13]. We extracted data on the intervention costs and the economic benefits following the intervention. When economic consequences of the intervention were described at several points in time, we used the longest follow up in the primary analysis. Base case costs were used if sensitivity analyses were performed. We extracted all direct and indirect costs of the intervention as well as savings to the extent that they were reported. As only some studies identified indirect costs, our analysis is limited to direct costs.

In cases of missing information concerning inflation adjustment, we assumed that the costs were adjusted to the last year of the study period. Moreover, if a low/high range of intervention costs or savings was reported, we used the average cost for our analysis.

### Quality Assessment

The methodological quality of the economic evaluations was assessed based on the methodology checklist recommended by SIGN (Scottish Intercollegiate Guidelines Network). Additionally, for an overall assessment of studies we answered one question from selection 2 of the SIGN statement, “How well was the study conducted?”, with the following coding: “++” denotes that all or most of the criteria have been fulfilled, “+”that some of the criteria have been fulfilled, and “−” that few or no criteria were fulfilled [14].

We used the Consolidated Health Economic Evaluation Reporting Standards (CHEERS) statement to assess the reporting quality of studies [15], although one of the CHEERS criteria was not relevant for our systematic review because of the selection criteria. Each CHEERS criterion was assigned a weight ranging from one to three (representing studies that reported well, reported poorly or did not report).

### Analysis of Results

Intervention costs and cost savings following the intervention were recalculated as costs per month during the intervention period and during the length of follow-up, respectively. We estimated the savings-to-cost ratio and the save–cost difference adjusted to 2013 US$. A savings-to-cost ratio larger than 1 indicated savings exceeding costs, and a positive save–cost difference value indicated net savings. Both intervention costs and cost savings were adjusted to 2013 values using an annual country-specific consumer price index [16]. After adjustment, these values were converted to 2013 US$ using the purchasing power parity (PPP) conversion factor [17]. We computed the summary median cost, cost savings per month, savings-to-cost ratio and difference values per month with a minimum–maximum range. We analyzed the effects of study characteristics on the cost-benefit results. We assumed that heterogeneity could have been due to the types of HAIs studied, the intervention duration, the hospital sizes, the number of patients, the target populations and the varying levels of methodological quality among these economic evaluations. All analyses were performed in Microsoft Excel (2010 Microsoft Corporation).

## Results

### Literature Search

**Fig 1:**
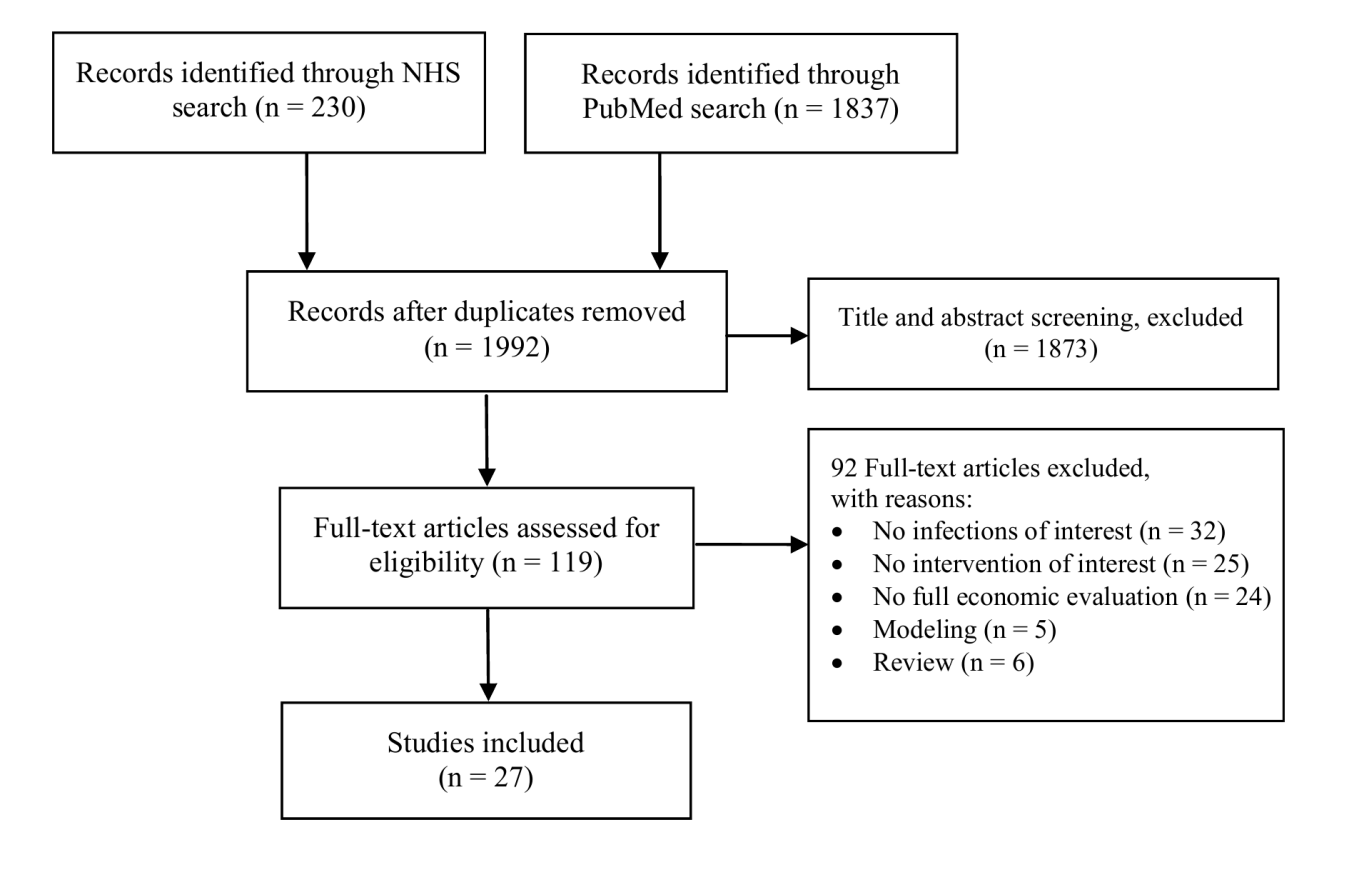
Flow diagram for the systematic review process to select studies.

Through our searches, a total of 1992 potentially relevant citations were identified, of which twenty-seven articles fulfilled the inclusion criteria [18–44]. A flow diagram of the search and selection strategy is shown in Fig 1. Excluded studies are listed in S4 Table.

### Study Descriptions

Most of the studies were performed in North America or Europe (n = 19, 70.4%). Approximately 63% of identified studies on the prevention of HAIs were performed in surgical departments (n = 10, 37.0%) or intensive care units (n = 7, 25.9%). The studies employed several methods to calculate effectiveness; 37.0% were randomized control studies (n = 10) and 63.0% were quasi-experimental (n = 17). More than half of the studies (n = 15, 55.6%) stated that they used the definitions of HAIs provided by the Centers for Disease Control and Prevention (CDC). Five studies focused on more than one site of HAI (18.5%). Most interventions aimed to prevent HAIs by reducing person-to-person transmission (n = 7, 25.9%), primarily by hand decontamination (S3 Table). The most frequent method used to prevent SSIs was optimal antibiotic prophylaxis (n = 6, 75.0%). Education programs were included in 8 studies (29.6%). Most interventions lasted for more than one year (n = 16, 59.3%), and only 6 studies performed a sensitivity analysis (22.2%). CEAs and CBAs were the most common types of economic evaluations. Only two of the studies were not from the healthcare provider cost perspective (7.4%), although most of the studies did not report the cost perspective they adopted (n = 18, 66.7%). The majority of studies used data collection to obtain cost data (n = 15, 55.6%). Resource costs were calculated using micro-costing, charges and mixed models. Many of the studies were funded by government sources (n = 11, 40.7%), although several studies did not include a statement on funding (n = 10, 37.0%). Further characteristics of the included publications are listed in Table 1.

**Table 1:** Characteristics of the studies.

Descriptive characteristics		Number (%)	Descriptive characteristics		Number (%)
**Geographical region of study**	United States/Canada	10 (37.0)	Type of intervention^*^	Hand hygiene	6 (22.2)
	Europe	9 (33.3)		Aseptic technique at insertion	4 (14.8)
	Asia	7 (25.9)		Optimal antibiotic prophylaxis	6 (22.2)
	Africa	1 (3.7)		Aseptic intubation and suctioning	2 (7.4)
**Target population/setting**	Intensive care unit	7 (25.9)		Limit duration of catheter	1(3.7)
	Surgery	11 (40.7)		Local skin preparation (catheters)	2 (7.4)
	Pediatric	1 (3.7)		Educational program	8 (29.6)
	Other patients or setting	5 (18.5)		Other interventions	20 (70.0)
	Hospital wide	3 (11.1)	**Type of economic evaluation**	CBA	9 (33.3)
**Gender**	Male	0 (0.0)		CEA	9 (33.3)
	Female	1 (3.7)		CMA	4 (14.8)
	Both	19 (70.4)		CEA+CBA	4 (14.8)
	Not stated	7 (25.9)		CEA+CUA	1 (3.7)
**Age group**	Children	1 (4)	**Cost perspective**	Healthcare provider	7 (25.9)
	Adult	4 (15)		Other perspective	2 (7.4)
	Mixed	13 (48)		Not stated	18 (66.7)
	Not stated	7 (26)	**Source of cost data**	Data collection	15 (55.6)
	Other (0–25, Patients ≥16)	2 (7)		Database	4 (14.8)
**Type of HAIs**	Surgical site infection	8 (29.7)		Mixed	8 (29.6)
	Urinary tract infection	4 (14.8)	**Source of effectiveness data**	Pre-post	8 (29.6)
	Bloodstream infection	5 (18.5)		Cohort	5 (18.5)
	Pneumonia	3 (11.1)		Randomized control trial	10 (37.0)
	More than one site of HAI	5 (18.5)		Case-control	1 (3.7)
	HAIs in general	2 (7.4)		Other quasi experimental design	3 (11.1)
**HAI definition**	CDC	15 (55.6)	**Method of cost calculation**	Accounting	2 (7.4)
	Other standard	5 (18.5)		Charges	7 (25.9)
	Not stated	7 (25.9)		Cost-to-charge-ratio	1 (3.7)
**Duration of intervention**	Six months or less	5 (18.5)		Micro-costing	6 (22.2)
	7–12 months	5 (18.5)		Mixed	7 (25.9)
	13–24 months	10 (37.0)		Other methods	4 (14.8)
	More than two years	6 (22.2)	**Discounting**	Yes	7 (25.9)
	Not stated	1 (3.7)		Not stated	14 (51.9)
**Sensitivity analysis**	Yes	6 (22.2)		Not necessary	6 (22.2)
	No	21 (77.8)			

HAI, hospital acquired infections; CDC, Centers for Disease Control and Prevention; CEA, Cost-effectiveness analysis; CBA, cost-benefit analysis; CMA, cost-minimization analysis; CUA, cost-utility analysis.

* Many of included studies used more than one type of intervention. Proportion based on total number of included studies.

### Assessment of Reporting Quality

We assessed the reporting quality of 26 studies using the CHEERS statement. Of these, we were able to identify 13 studies as economic evaluations based on the title (50.0%). Most articles presented a clear study question and an explicit statement of the background for the study (n = 19, 73.1%). Studies generally reported the target population and subgroups well (n = 16, 61.5%). Statements regarding the perspective of the study and its relation to costs were missing in 17 studies (65.4%). The majority of studies did not include a statement on the choice of discount rates (n = 11, 42.3%) or included poor reporting on the choice (n = 6, 23.1%). Only 9 studies properly described what approaches were used to estimate resources and costs (34.6%). A price date, the method of price adjustment and the currency used were not noted in 10 studies (38.5%). Half of the selected studies did not report conflicts of interest (Table 2).

**Table 2:** Assessment of the reporting quality of included studies using CHEERS statement^a^.

Section/item		Number of studies (%) reporting		
		well	poorly	not
**Title and abstract Introduction Methods**	Title	9 (34.6)	4 (15.4)	13 (50.0)
	Abstract	8 (30.8)	15 (57.7)	3 (11.5)
	Background and objectives	19 (73.1)	7 (26.9)	0 (0.0)
	Target population and subgroups	16 (61.5)	7 (26.9)	3 (11.5)
	Setting and location	18 (69.2)	5 (19.2)	3 (11.5)
	Study perspective	7 (26.9)	2 (7.7)	17 (65.4)
	Comparators	22 (84.6)	4 (15.4)	0 (0.0)
	Time horizon	20 (76.9)	5 (19.2)	1 (3.8)
	Discount rate	9 (34.6)	6 (23.1)	11 (42.3)
	Choice of health outcomes	18 (69.2)	7 (26.9)	1 (3.8)
	Measurement of effectiveness	17 (65.4)	8 (30.8)	1 (3.8)
	Measurement and valuation of preference based outcomes	16 (61.5)	6 (23.1)	4 (15.4)
	Estimating resources and costs	9 (34.6)	10 (38.5)	7 (26.9)
	Currency, price date, and conversion	13 (50.0)	3 (11.5)	10 (38.5)
	Assumptions	5 (19.2)	17 (65.4)	4 (15.4)
	Analytical methods	13 (50.0)	6 (23.1)	7 (26.9)
**Results**	Study parameters	18 (69.2)	4 (15.4)	4 (15.4)
	Incremental costs and outcomes	9 (34.6)	10 (38.5)	7 (26.9)
	Characterizing uncertainty	2 (7.7)	10 (38.5)	14 (53.8)
	Characterizing heterogeneity	10 (38.5)	10 (38.5)	6 (23.1)
**Discussion**	Study findings, limitations, generalizability, and current knowledge	13 (50.0)	10 (38.5)	3 (11.5)
**Other**	Source of funding	16 (61.5)	0 (0.0)	10 (38.5)
	Conflicts of interest	13 (50.0)	0 (0.0)	13 (50.0)

a. Pickard et al. excluded from the reporting quality section

### Assessment of Method Quality

**Fig 2:**
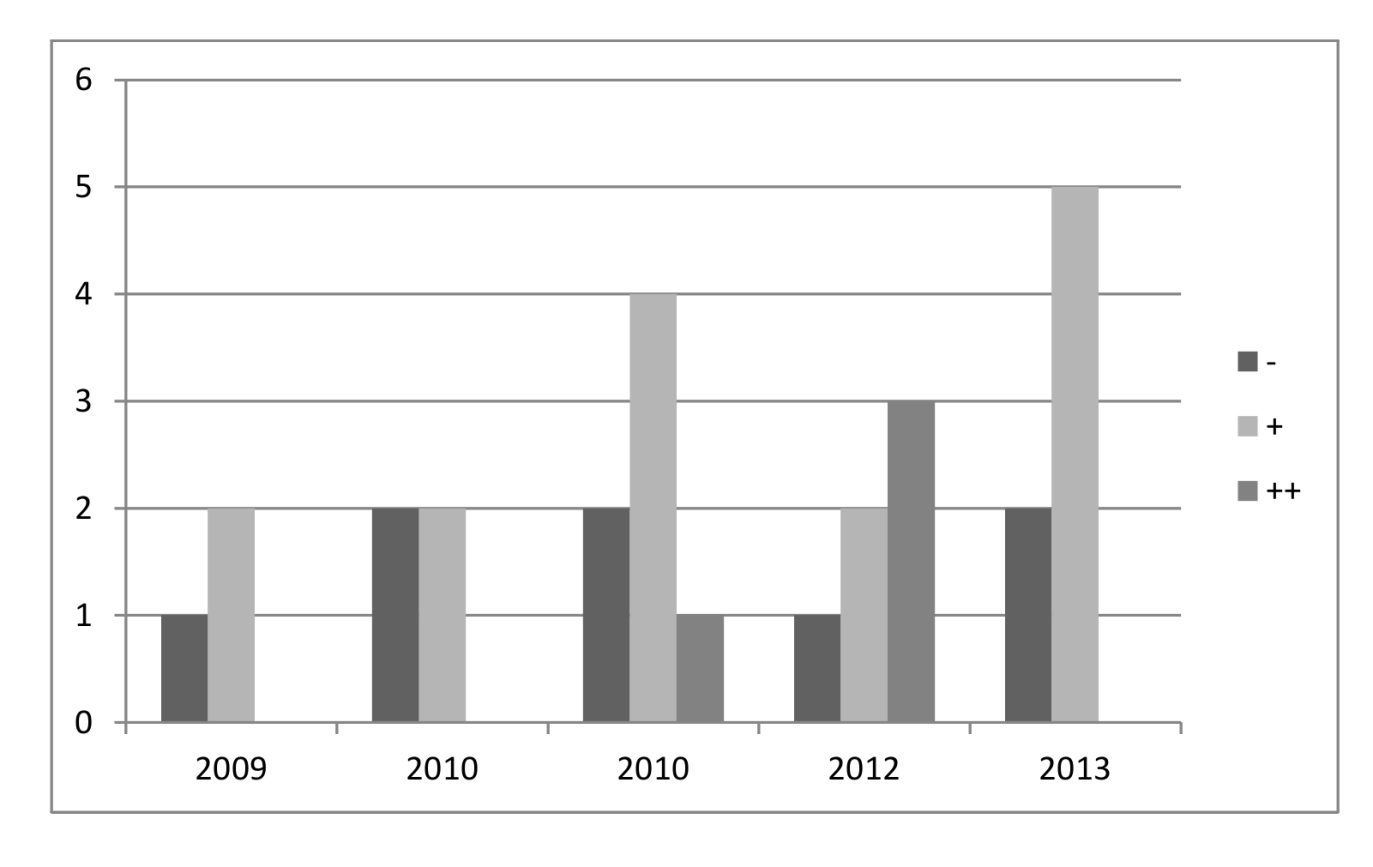
Methodological quality of studies and publication year.

Most of the 27 economic studies included in the method quality assessment defined an answerable study question (n = 14, 51.9%), but few papers included all costs relevant to the viewpoint of the study (Table 3). In the overall method quality assessment, 4 studies (14.8%) were evaluated as “++”, 15 (55.5%) as “+” and 8 (29.6%) as “–”. Since 2009, the quantity of publications regarding prevention programs for HAIs has increased, while the quality of these studies has not improved, as shown in Fig 2, which illustrates the method quality for different publication years.

**Table 3:** Assessment of method quality of included studies using SIGN guideline.

In a well conducted economic study. . .	Well covered (%)	Adequately addressed (%)	Poorly addressed (%)	Not addressed (%)	Not reported (%)	Not applicable (%)
There is a defined and answerable study question	14 (51.9)	7 (25.9)	4 (14.8)	2 (7.4)	–	–
The economic importance of the question is clear	6 (22.2)	6 (22.2)	6 (22.2)	7 (25.9)	2 (7.4)	–
The choice of study design is justified	4 (14.8)	14 (51.9)	9 (33.3)	–	–	–
All costs that are relevant from the viewpoint of the study are included and are measured and valued appropriately	2 (7.4)	8 (29.6)	11 (40.7)	6 (22.2)	–	–
The outcome measures used to answer the study question are relevant to that purpose and are measured and valued appropriately	3 (11.1)	18 (66.7)	6 (22.2)	–	–	–
If discounting of future costs and outcomes is necessary, it has been performed correctly	4 (14.8)	2 (7.4)	1 (3.7)	6 (22.2)	8 (29.6)	6 (22.2)
Assumptions are made explicit, and a sensitivity analysis performed	4 (14.8)	3 (11.1)	3 (11.1)	16 (59.3)	1 (3.7)	–
The decision rule is made explicit, and comparisons are made on the basis of incremental costs and outcomes	1 (3.7)	8 (29.6)	9 (33.3)	7 (25.9)	2 (7.4)	–
If discounting of future costs and outcomes is necessary, it has been performed correctly	3 (11.1)	11 (40.7)	13 (48.1)	–	–	–

### Cost-Benefit Analysis

Common cost components included nurse/physician time, antimicrobials, administration costs and pharmaceuticals. Intervention costs were usually reported as the global costs of the intervention (n = 9, 33.3%). Extra costs of an HAI and costs of extra hospitalization days due to HAIs were the major cost components used for cost savings calculations.

The median savings-to-cost ratio across the 18 studies reporting both values was US $7.0 (IQR 4.2–30.9), and the median net global saving of the 19 studies was US $13,179 (IQR 5,106–65,850) per month. The median cost across the 20 studies reporting intervention costs was US $1,114 (IQR 174–6234) per month. The median saving across the 24 studies reporting this figure was US $12,519 (IQR 6,273–65,309) per month. Most of the 18 articles reporting both intervention and saved costs calculated a savings-to-cost ratio >1 and a positive save–cost difference, and only one study showed that infection control interventions were not economically justified because the savings-to-cost ratio was < 1 or the save–cost difference was negative (Table 4).

**Table 4:** Intervention costs and cost savings.

Study ID		Country	Intervention target	Duration ofintervention	Total number of patients	Total intervention cost^a^	Total cost savings^a^	savings-to-cost ratio	Save–cost difference^a^	Intervention efficacy^b^
Burden[18]		USA	ICU, 24 beds	24 months	6,059	5,567	23,306	4.2	17,739	Yes
Chen[19]		Taiwan	Hospital-wide, 2,200 beds	45 months	552,146	353	8,376	23.7	8,023	Yes
Clarke[20]		USA	Hospital-wide, 276 beds	19 months	2,228	2,130	6,742	3.2	4,612	Yes
Cohen[21]		USA	MICU, 20 beds	12 months	477	10,072	73,760	7.3	63,688	Yes
Dijksman[22]		Netherlands	Elective gastrointestinal surgery	12 months	289	2,318	45,892	19.8	43,573	Yes
Fraher[23]		Ireland	Hospital-wide, 535 beds	12 years	1,932	6,137	14,611	2.4	8,474	Yes
Harris[26]		USA	Pediatric ICU, 20 beds	9 months	2,379	702	889,697	1,267.4	888,995	Yes
Nakamura[30]		Japan	Surgical ward, 7,500 surgeries yearly	24 months	410	93	3,541	38.2	3,448	Yes
Perez[32]		Spain	Heart surgery, 500 heart surgery yearly	23 months	1,399	916	6,022	6.6	5,106	Yes
Pickard [33]	Nitrofurazone	UK	Catheterization patients, 24 hospitals	1,5 months	2,153	10,127	293,981	29.0	283,854	Yes
	Silver alloy	UK	Catheterization patients, 24 hospitals	1,5 months	2097	12,599	78,449	6.2	65,850	No
Piednoir[34]		France	ICU, 8 beds	24 months	919	133	7,195	53.9	7,062	Yes
Raschka[35]		Canada	Six acute care, 82,046 admissions yearly	48 months	NS	115,269	626,850	5.4	511,581	NS
Schwebel[36];	CHGIS dressing &3 days change	France	Seven ICUs	19 months	818	1,522	10,186	6.7	8,664	Yes
	CHGIS dressing &7 days change	France	Seven ICUs	19 months	818	1,114	4,536	4.1	3,422	Yes
Singh[37]		India	Cardiovascular surgical unit, 68 beds	4 months	2,838	589	21,468	36.5	20,879	Yes
Sona[38]		USA	ICU, 24 beds	12 months	1,648	215	68,775	320.5	68,560	Yes
Speroni[39]		USA	Ventilation Patients,155 beds	13 months	154	76	1,290	17.0	1,214	No
van den Broek[41]		Netherlands	10 hospitals	5 months	2,943	6,331	1,837	0.3	-4,494	No
Waters[42]		USA	103 ICU, 6 hospitals	4 months	NS	43,628	114,422	2.6	70,794	Yes
Gulluoglu[24]		Turkey	Surgical ward	6.5 years	369	NS	9	NA	NA	Yes
Halleberg Nyman[25]		Sweden	Orthopedic surgical ward	21 months	170	NA	1,130	NA	NA	No
Liau[27]		Singapore	Surgical ward	24 months	2,408	NS	12,057	NA	NA	Yes
Mathur[28]		India	Surgical ward	20 months	197	NA	616	NA	NA	No
Mian[29]		USA	Hematology-oncology unit	36 months	NS	NA	38,032	NA	NA	Yes
Nthumba[31]		Kenya	Surgical ward, 5000 procedures yearly	2 months	3,133	8	NS	NA	NA	No
Teshima[40]		Japan	Surgical ward	48 months	253	16–25	NS	NA	NA	Yes
Weight [43]		USA	Pediatric ward	NS	3600	NA	NS	NA	NA	No
Zhou[44]		China	Surgical ward	7 months	614	20,949–30,419	3,709	NA	13,179	No

NS, Not Stated; NA, Not Applicable; CHGIS, chlorhexidine gluconate-impregnated sponge; ICU, Intensive Care Unit; MICU, Medical Intensive Care Unit

a. Cost per month in 2013 US$.

b. Intervention reported to be statistically significantly efficacious.

The effects of study characteristics on the savings-to-cost ratios and net savings were very diverse. Higher savings-to-cost ratios were observed in studies that focused on pneumonia prevention compared with prevention programs focusing on other infections. However, the studies that considered several types of infections in their infection prevention program calculated a higher savings-to-cost ratio compared with studies dedicated to a single type of HAI. Savings-to-cost ratios were higher in studies with smaller number of patients. Larger hospitals (>500 beds compared with smaller hospitals) with shorter intervention durations (≤12 months compared with longer durations) exhibited higher savings-to-cost ratios. Nevertheless, savings-to-cost ratios were considerably lower in multi-center studies. Programs for prevention of HAIs in surgical units have higher savings-to-cost ratios compared with prevention programs for HAIs in intensive care units or in hospital-wide studies. Savings-to-cost ratios were much higher in studies that did not report or poorly addressed costs and had inappropriate measurement and evaluation, compared with studies addressing these factors adequately or well. This result shows that studies without complete identification and measurement of relevant costs overestimate the savings-to-cost ratios. We found similar savings-to-cost ratios among studies that were assigned an overall assessment of “-” (Table 5).

**Table 5:** Effects of study characteristics on results^a^.

Characteristics		intervention cost	cost savings	savings-to-cost ratio	save–cost difference
**Types of HAIs**	Surgical site infection	50 (8–93), n = 2	3,541 (9–12057), n = 5	38.2, n = 1	8,313 (3448–13,179), n = 2
	Urinary tract infection	8,229 (2,130–12,599), n = 3	6,742 (1,130–293,981), n = 4	4.7 (0.3–29.0), n = 3	35,231 (-4,494–283,854), n = 3
	Bloodstream infection	5,567 (1,114–10,072), n = 4	12,399 (616–73,760), n = 5	4.2 (2.4–7.3), n = 4	8,664 (3,422–63,688), n = 4
	Pneumonia	215 (76–916), n = 3	6,022 (1,290–68,775), n = 3	17 (6.6–320.5), n = 3	5,106 (1,214–68,560), n = 3
	More than one site of HAI	702 (133–43,628), n = 5	45,892 (7,195–889,697), n = 5	36.5 (2.6–1,267.4), n = 5	43,573 (7,062–888,995), n = 5
	HAIs in general	57,811 (353–115,269), n = 2	317,613 (8,376–626,850), n = 2	14.6 (5.4–23.7), n = 2	259,802 (8,023–511,581), n = 2
**Intervention duration**	≤ 12 months	4,325 (8–43,628), n = 9	71,267 (1,837–889,697), n = 9	19.8 (0.3–1267.4), n = 8	64,769 (-4,494–888,995), n = 9
	> 12 months	1,114 (76–115,269), n = 10	6,969 (9–626,850), n = 15	6.6 (2.4–53.9), n = 10	7,062 (1,214–511,581), n = 10
**Hospital size**	≤500 beds	389 (8–2,130), n = 4	4,016 (1,130–889,697), n = 4	17.0 (3.2–1267.4), n = 3	4,612 (1,214–888,995), n = 3
	>500beds	752 (93–10,072), n = 10	11,494 (9–889,697), n = 14	21.7 (2.4–320.5), n = 10	13,179 (3,448–68,560), n = 11
	Several hospitals	10,127 (1,114–115,269), n = 5	78,449 (1,837–626,850), n = 5	5.4 (0.3–29.0), n = 5	65,850 (-4,494–511,581), n = 5
**Number of Patients**	≤1000	1,114 (76–10,072), n = 6	3,709 (9–73,760), n = 10	17.0 (4.1–53.9), n = 6	7,863 (1,214–63,688), n = 7
	1000–3000	2,130 (215–12,599), n = 8	18,039 (1,837–889,697), n = 9	6.6 (0.3–1267.4), n = 8	20,879 (-4,494–888,995), n = 8
	>3000	353 (8–5,567), n = 3	15,841(8,376–23,306), n = 2	13.9 (4.2–23.7), n = 2	12,881 (8,023–17,739), n = 2
**Target population**	Intensive care unit	1,318 (133–43,628), n = 7	46,040 (4,536–889,697), n = 7	7.0 (2.6–1,267.4), n = 7	40,714 (3,422–888,995), n = 7
	Surgery	589 (8–2,318), n = 5	3,709 (9–45,892), n = 9	28.1 (6.6–38.2), n = 4	13,179 (3,448–43,573), n = 5
	Hospital-wide	2,130 (353–6,137), n = 3	8,376 (6,742–14,611), n = 3	3.2 (2.4–23.7), n = 3	8,023 (4,612–8,474), n = 3
	Other patients or setting	8,229 (76–12,599), n = 3	38,032 (1,290–293,981), n = 4	11.6 (0.3–29.0), n = 3	33,532 (-4,494–283,854), n = 3
**All costs are included andare measured and valued appropriately (SIGN)**	Well covered	5,825 (1,114–12,599), n = 2	44,317 (4,536–293,981), n = 2	6.5 (4.1–29.0), n = 2	37,257 (3,422–283,854), n = 2
	Adequately addressed	2,318 (353–115,269), n = 7	14,922 (1,837–626,850), n = 8	5.4 (0.3–36.5), n = 7	17,029 (-4,494–511,581), n = 8
	Poorly or Not addressed	458 (8–10,072), n = 10	9,626 (9–889,697), n = 14	17.0 (2.4–1,267.4), n = 9	8,474 (1,214–888,995), n = 9
**How well was the study conducted? (SIGN)**	++	1,920 (353–12,599), n = 4	28,039 (4,536–293,981), n = 4	13.2 (4.1–29.0), n = 4	26,119 (3,422–283,854), n = 4
	+	2,130 (93–115,269), n = 11	16,762 (1,130–889,697), n = 14	6.6 (0.3–1,267.4), n = 11	19,309 (-4,494–888,995), n = 12
	-	105 (8–6,137), n = 4	4,243 (9–38,032), n = 6	17.0 (2.4–53.9), n = 3	7,062 (1,214–8,474), n = 3

HAI, Hospital acquired infections.

a. Median (Range) value, cost per month in 2013 US$. n, number of studies.

## Discussion

We systematically reviewed and assessed the quality of the methods and reporting of selected economic evaluation studies regarding HAI prevention interventions and their economic benefits. Prevention interventions for HAIs were reported as statistically significantly efficacious in many studies, and our analysis shows that these interventions have significant economic benefits. On average, the savings of a prevention program were 11 times greater than the costs. The highest save–cost difference was identified in a study by Harris et al., which used improvement practices of hand hygiene, oral care and central-line catheter care in a single hospital [26]. Only Van den Broek et al. calculated a negative save–cost difference for a program intended to reduce the use of urethral catheters through an implementation strategy focused on a limited number of recommendations from the Dutch Working Party on Infection Prevention (WIP) guideline [41]. Nevertheless, it should be noted here that the most important aspect of an infection prevention program is reduction of harm and loss of life. Although the hospital-wide hand decontamination had a high cost savings benefit, only one of four hand hygiene programs was hospital wide, while 3 were implemented in intensive care units.

Interventions targeting several types of HAIs or HAIs in general were associated with higher economic benefits than prevention interventions for a single type of infection. A strong association was identified between intervention duration and cost benefit. The median save–cost difference among studies with intervention periods of 12 months or less was 9 times greater than that among studies with longer intervention durations. Higher savings-to-cost ratios were found in larger hospitals. These ratios were very low in multi-center studies; however, the median save–cost difference in such studies was high. Multi-center trials are usually very expensive to implement and much more complex than single-center studies. Nevertheless, multi-center prevention programs exhibit a better net-savings than single-center programs.

The quality of reporting in the studies was low. Only fourteen articles reported more than half of the CHEERS items appropriately, and only seven studies reported 70% or more of these items well. We found similar results in our assessment of methodological quality. Various studies had low internal validity; for example, several studies did not include some of the relevant costs in the economic evaluation that could have a substantial impact on the results. Discounting future costs and outcomes was necessary in most of the selected studies; nevertheless, such discounting was often not performed. The quality of economic studies is directly related to the presence of a sensitivity analysis. Therefore, the Oxford Centre for Evidence-based Medicine (OCEBM) classified economic studies without a sensitivity analysis as level IV evidence [45]. Nevertheless, few studies in our review performed a sensitivity analysis of their economic evaluation.

Higher quality studies fulfilling all or most of the methodology criteria have higher savings-to-cost ratios compared with studies of intermediate quality, but the savings-to-cost ratios in low-quality studies fulfilling few or no criteria were extremely large. This may represent overestimation due to inappropriate designs for economic evaluations or the failure to consider some relevant costs. Low-quality clinical trials inflated the estimated treatment efficacy by 30–50%, according Moher et al. [46]. An overestimation of benefit-cost ratio may therefore exist in low-quality economic evaluations.

Only a small number of systematic reviews examined the economic impact of interventions for HAI prevention [9,12,13]. Farbman et al. [13] focused on economic evaluations of infection control interventions targeting methicillin-resistant *Staphylococcus aureus* (MRSA) in a study that was methodologically similar to our systematic review. They found that the median savings-to-cost ratio among 18 MRSA studies was US $7.16 (IQR 1.37–16), which is similar to the present findings. Their study observed that interventions with longer durations (>6 months) had higher savings-to-cost ratios compared with interventions of shorter duration. In contrast, the present study found higher savings-to-cost ratios for interventions with a duration of 12 months or shorter compared with interventions with longer durations.

Our study has some limitations, as is true of all systematic reviews of economic evaluations. Due to the wide variety of terminology in the fields of full economic evaluations, hospital acquired infections and prevention interventions, some relevant articles may have been missed in our review of the literature. However, we used two large literature databases, and a variety of keywords were matched to database-specific indexing terms. In this review, databases were searched based on the English and German languages. Therefore, publications in other languages were not detected using this search strategy. Because various types of interventions and various combinations of interventions were included and because some intervention cost components were lacking, we could not classify the interventions’ effects with respect to economic benefits. The distribution of cost values was not normal, and the homogeneity of costs was not high; therefore, we were unable to perform a formal meta-analysis. One further limitation of our study is a potential publication bias. Studies with a negative save to cost ratio are unlikely to get published compared to studies with a positive result and these studies are not included in our review.

In conclusion, the included studies indicate that HAI prevention interventions yield very positive cost-benefit estimations. The quality of economic evaluations should be improved to provide better information to healthcare policy makers and clinicians. International standardization of cost estimations for HAIs would enable economic evaluation studies to perform more precise assessments of economic benefits and cost changes associated with HAI prevention programs.

## Supporting Information

S1 Table PRISMA Checklist. (PDF)

S2 Table PubMed Search Strategy. (PDF)

S3 Table Hospital acquired infections interventions in individual studies. (PDF)

S4 Table Excluded studies with reason. (PDF)
