# Supplementary material for: Economic Evaluation of Interventions for Prevention of Hospital Acquired Infections: A Systematic Review
Source: PLoS One. 2016 Jan 5;11(1):e0146381. doi: 10.1371/journal.pone.0146381 (PMC4701449; doi:10.1371/journal.pone.0146381)
Supplement: S2 Table — (PDF) [file pone.0146381.s002.pdf]

S2 Table. PubMed Search Strategy

(prevention) OR (health promotion) OR ("primary prevention"[Mesh]) OR (lifestyle) OR (intervention) OR (counseling) OR (multimodal) OR (multi-factor*) OR (multifactor*) OR (multi-component) OR (risk assessment) OR (risk factor*) OR (health education) OR ("health behavior"[Mesh]) OR ("risk reduction behavior"[Mesh]) OR ("risk factors"[Mesh]) AND ("Economics"[Mesh]) OR (Economics) OR (economic*) OR ("Costs and Cost Analysis"[Mesh]) OR (cost) OR (efficiency) OR (return on investment) OR (direct cost*) OR (indirect cost*) OR (economic evaluation*[ti]) OR (economic analy*[ti]) OR (cost analy*[ti]) OR (cost effectiveness[ti]) OR (cost benefit*[ti]) OR (cost utilit*[ti])) AND (nosocomial infection) OR (nosocomial) OR (hospital-acquired) OR (healthcare-associated) OR (hospital infection) OR ("Cross Infection"[Mesh])Filters: Publication date from 2009/01/01 to 2014/01/01; Humans; English; German
