# Supplementary material for: Economic Evaluation of Interventions for Prevention of Hospital Acquired Infections: A Systematic Review
Source: PLoS One. 2016 Jan 5;11(1):e0146381. doi: 10.1371/journal.pone.0146381 (PMC4701449; doi:10.1371/journal.pone.0146381)
Supplement: S3 Table — (PDF) [file pone.0146381.s003.pdf]

S3 Table. Hospital acquired infection interventions in individual studies

Study ID	Interventions
Burden[14]	A mandatory simulation-based program to teach central venous catheter insertion
Chen[15]	Hand hygiene promotion program; multidisciplinary approach involving cognition, equipment, and behavior
Clarke[16]	Exclusive use of silver alloy catheters, limit the movement of the catheter after insertion, repositioning of the catheter tubing, removal of the indwelling urinary catheter
Cohen[17]	Simulation-based education intervention in central venous catheter insertion
Dijksman[18]	Perioperative selective decontamination of the digestive tract
Fraher[19]	Total parenteral nutrition surveillance clinical nurse manager
Gulluoglu[20]	Prophylactic antibiotic administration
Halleberg Nyman[21]	Intermittent or indwelling urinary catheterisation
Harris[22]	Improving practices of hand hygiene, oral care and central-line catheter use
Liau[23]	Hair removal, antibiotic administration, glucose and temperature monitoring
Mathur[24]	Perioperative antibiotic prophylactic regimen
Mian[25]	Quality improvement: education, CVL maintenance care bundles, CVL insertion guideline
Nakamura[26]	Triclosan-coated polyglactin suture materials with antimicrobial activity
Nthumba[27]	Plain soap and water with alcohol-based handrub
Perez Granda[28]	Routine introduction of aspiration of subglottic secretions in patients after major heart surgery
Pickard[29]	Antimicrobial-impregnated silicone catheter (nitrofurazone) and antiseptic-coated hydrogel latex catheter (silver alloy) were compared to standard polytetrafluoroethylene-coated catheters
Piednoir[30]	Promotion of contact precautions and hand rubbing by additional training sessions, antibiotic policy and practices, systematic screening on admission by rectal swab
Raschka[31]	Reduction of bacteremias, control of MRSA, hand hygiene program, *Clostridium difficile* isolation and treatment guidelines, CAUTI prevention initiatives
Schwebel[32]	CHGIS versus no CHGIS (standard dressing), seven-day dressing change was compared with three-day dressing change
Singh[33]	Two-module training step-by-step teaching program
Sona[34]	A simple oral care protocol to assist in prevention of bacterial growth of plaque by cleaning the patients' teeth
Speroni[35]	Continuous subglottic suctioning endotracheal tubes versus standard endotracheal tubes among intubated patients
Teshima[36]	New hydrocolloid dressing (Karayahesive) or a polyurethane foam dressing (Tegaderm plus Pad) after sternal wound closure
Van den Broek[37]	Reduction of the use of urinary catheters
Waters[38]	Comprehensive Unit-Based Safety Program (improve safety culture, teamwork, and communication), evidence-based care to reduce CLABSIs and VAP
Weight[39]	Avagard (waterless, scrubless, and brushless hand antiseptic) was compared with traditional pre-surgical antiseptic-impregnated hand brush for hand scrubbing
Zhou[40]	A guideline for the appropriate use of antibiotics

CVL, central venous line; MRSA, methicillin-resistant Staphylococcus aureus; CAUTI, catheter-associated urinary tract infection; CHGIS, chlorhexidine gluconate-impregnated sponge; CLABSIs, central line–associated bloodstream infections; VAP, ventilator-associated pneumonia
