# Supplementary material for: Economic Evaluation of Interventions for Prevention of Hospital Acquired Infections: A Systematic Review
Source: PLoS One. 2016 Jan 5;11(1):e0146381. doi: 10.1371/journal.pone.0146381 (PMC4701449; doi:10.1371/journal.pone.0146381)
Supplement: S4 Table — (PDF) [file pone.0146381.s004.pdf]

S4 Table. **Excluded studies with reason**

	Study ID	Title	Reasons for exclusion
1	Adibi et al. (2013)	Reduction in hospital admission rates due to post-prostate biopsy infections after augmenting standard antibiotic prophylaxis	• No intervention of interest
2	Ahmed et al. (2012)	Catheter-associated bloodstream infection in the pediatric intensive care unit: a multidisciplinary approach	• No full economic evaluation
3	Al-Badriyeh et al. (2009)	Cost-effectiveness evaluation of voriconazole versus liposomal amphotericin B as empirical therapy for febrile neutropenia in Australia	• No intervention of interest
4	Ali et al. (2012)	Clostridium difficile infection in hospitalized liver transplant patients: a nationwide analysis	• No full economic evaluation<br>• No infection of interest
5	Ali et al. (2012)	Effect of surface coating and finish upon the cleanability of bed rails and the spread of Staphylococcus aureus	• No full economic evaluation<br>• No infection of interest
6	Al-Tawfiq et al. (2010)	Decreasing ventilator-associated pneumonia in adult intensive care units using the Institute for Healthcare Improvement bundle	• No intervention of interest<br>• No full economic evaluation
7	Anderson et al. (2011)	The network approach for prevention of healthcare-associated infections: long-term effect of participation in the Duke Infection Control Outreach Network	• No intervention of interest
8	Apisarnthanarak et al. (2010)	Reduction of seasonal influenza transmission among healthcare workers in an intensive care unit: a 4-year intervention study in Thailand	• No infection of interest
9	Apisarnthanarak et al. (2010)	Impact of education and an antifungal stewardship program for candidiasis at a Thai tertiary care center	• No infection of interest
10	Attenello et al. (2010)	Hospital costs associated with shunt infections in patients receiving antibiotic-impregnated shunt catheters versus standard shunt catheters	• No intervention of interest
11	Bailey et al. (2011)	Economic value of dispensing home-based preoperative chlorhexidine bathing cloths to prevent surgical site infection	• Modeling
12	Balegar et al. (2013)	Extending total parenteral nutrition hang time in the neonatal intensive care unit: is it safe and cost effective?	• No full economic evaluation<br>• No intervention of interest
13	Barbut et al. (2012)	New molecular methods for the diagnosis of Clostridium difficile infections	• Review
14	Barsanti et al. (2009)	Infection prevention in the intensive care unit	• Review
15	Bartsch et al. (2012)	The potential economic value of screening hospital admissions for Clostridium difficile	• No infection of interest
16	Baykasoglu et al. (2009)	Application of cost/benefit analysis for surgical gown and drape selection: a case study	• Modeling
17	Bilcke et al. (2009)	Cost-effectiveness of rotavirus vaccination: exploring caregiver(s) and "no medical care" disease impact in Belgium	• No infection of interest
18	Bird et al. (2010)	Adherence to ventilator-associated pneumonia bundle and incidence of ventilator-associated pneumonia in the surgical intensive care unit	• No full economic evaluation<br>• No intervention of interest
19	Blumenstein et al. (2012)	A glycerin hydrogel-based wound dressing prevents peristomal infections after percutaneous endoscopic gastrostomy (PEG): a prospective, randomized study	• No full economic evaluation
20	Boccalini et al. (2011)	Economic and clinical evaluation of a catch-up dose of 13-valent pneumococcal conjugate vaccine in children already immunized with three doses of the 7-valent vaccine in Italy	• No intervention of interest

21	Boyce et al. (2013)	Obtaining blood cultures by venipuncture versus from central lines: impact on blood culture contamination rates and potential effect on central line-associated bloodstream infection reporting	• No intervention of interest
22	Bukhari et al. (2012)	Application of ventilator care bundle and its impact on ventilator associated pneumonia incidence rate in the adult intensive care unit.	• No full economic evaluation
23	Byington et al. (2012)	Costs and infant outcomes after implementation of a care process model for febrile infants	• No infection of interest
24	Catanzaro et al. (2012)	Real-time polymerase chain reaction testing for Clostridium difficile reduces isolation time and improves patient management in a small community hospital	• No full economic evaluation<br>• No infection of interest
25	Ceppa et al. (2013)	Reducing surgical site infections in hepatopancreatobiliary surgery.	• No intervention of interest
26	Chenoweth et al. (2013)	Preventing catheter-associated urinary tract infections in the intensive care unit	• Review
27	Chow et al. (2012)	Effect of continuous oral suctioning on the development of ventilator-associated pneumonia: a pilot randomized controlled trial	• No full economic evaluation
28	Christopher et al. (2011)	Transmission dynamics of methicillin-resistant Staphylococcus aureus in a medical intensive care unit in India	• No intervention of interest<br>• No infection of interest<br>• No full economic evaluation
29	Courville et al. (2012)	Cost-effectiveness of preoperative nasal mupirocin treatment in preventing surgical site infection in patients undergoing total hip and knee arthroplasty: a cost-effectiveness analysis	• No intervention of interest
30	Cummings et al. (2010)	Hand hygiene noncompliance and the cost of hospital-acquired methicillin-resistant Staphylococcus aureus infection	• No infection of interest
31	Dancer et al. (2009)	Measuring the effect of enhanced cleaning in a UK hospital: a prospective cross-over study	• No infection of interest
32	Dendle et al. (2009)	Staphylococcus aureus bacteraemia as a quality indicator for hospital infection control	• No infection of interest
33	Doan et al. (2012)	Clinical and cost effectiveness of eight disinfection methods for terminal disinfection of hospital isolation rooms contaminated with Clostridium difficile 027	• No infection of interest
34	Dranitsaris et al. (2011)	Posaconazole versus fluconazole or itraconazole for prevention of invasive fungal infections in patients undergoing intensive cytotoxic therapy for acute myeloid leukemia or myelodysplasia: a cost effectiveness analysis	• No infection of interest
35	Eagye et al. (2009)	Surgical site infections: does inadequate antibiotic therapy affect patient outcomes?	• No intervention of interest
36	Forte et al. (2011)	Comparative cost-efficiency of the EVOTECH endoscope cleaner and reprocessor versus manual cleaning plus automated endoscope reprocessing in a real-world Canadian hospital endoscopy setting	• No intervention of interest
37	Gagne et al. (2010)	Systematic patients' hand disinfection: impact on meticillin-resistant Staphylococcus aureus infection rates in a community hospital	• No infection of interest
38	Gerber et al. (2013)	Identifying targets for antimicrobial stewardship in children's hospitals	• No full economic evaluation
39	Giglio et al. (2010)	Cost-effectiveness of the CRM-based 7-valent pneumococcal conjugated vaccine (PCV7) in Argentina	• No intervention of interest
40	Gray, M. (2010)	Reducing catheter-associated urinary tract infection in the critical care unit	• Review
41	Greer et al. (2009)	Keeping vulnerable children safe from pertussis: preventing nosocomial pertussis transmission in the neonatal intensive care unit	• No intervention of interest

42	Halton et al. (2010)	Cost-effectiveness of a central venous catheter care bundle	• Modeling
43	Hanmore et al. (2013)	Economic benefits of safety-engineered sharp devices in Belgium - a budget impact model	• No intervention of interest<br>• No infection of interest
44	Heimes et al. (2011)	Implementation and enforcement of ventilator-associated pneumonia prevention strategies in trauma patients	• No full economic evaluation
45	Hollenbeak et al. (2011)	Electronic measures of surgical site infection: implications for estimating risks and costs	• No full economic evaluation
46	Holmen Moller et al. (2012)	A cost-effectiveness analysis of reducing ventilator-associated pneumonia at a Danish ICU with ventilator bundle	• No intervention of interest
47	Holzmann-Pazgal et al. (2011)	Active surveillance culturing impacts methicillin-resistant Staphylococcus aureus acquisition in a pediatric intensive care unit	• No full economic evaluation<br>• No infection of interest
48	Huynh et al. (2013)	Plastic freezer bags: a cost-effective method to protect extraction sites in laparoscopic colorectal procedures?	• No intervention of interest
49	Illingworth et al. (2011)	Is closure of entire wards necessary to control norovirus outbreaks in hospital? Comparing the effectiveness of two infection control strategies	• No full economic evaluation<br>• No infection of interest
50	Johnson et al. (2010)	Preoperative chlorhexidine preparation and the incidence of surgical site infections after hip arthroplasty	• No full economic evaluation
51	Kaambwa et al. (2010)	Cost-effectiveness of rapid tests and other existing strategies for screening and management of early-onset group B streptococcus during labour	• No infection of interest
52	Kennedy et al. (2013)	Estimating hospital costs of catheter-associated urinary tract infection	• No intervention of interest
53	Landre-Peigne et al. (2011)	Efficacy of an infection control programme in reducing nosocomial bloodstream infections in a Senegalese neonatal unit	• No full economic evaluation
54	Lee at al. (2011)	The economic value of screening haemodialysis patients for methicillin-resistant Staphylococcus aureus in the USA	• No infection of interest
55	Lee at al. (2009)	Should vascular surgery patients be screened preoperatively for methicillin-resistant Staphylococcus aureus?	• No infection of interest
56	Lee at al. (2010)	The economic effect of screening orthopedic surgery patients preoperatively for methicillin-resistant Staphylococcus aureus	• No infection of interest
57	Lee at al. (2011)	Routine pre-cesarean Staphylococcus aureus screening and decolonization: a cost-effectiveness analysis	• No infection of interest
58	Leonhardt at al. (2011)	Clinical effectiveness and cost benefit of universal versus targeted methicillin-resistant Staphylococcus aureus screening upon admission in hospitals	• No infection of interest
59	Li et al. (2012)	Cost-effectiveness of supplementing a broth-enriched culture test with the Xpert meticillin-resistant Staphylococcus aureus (MRSA) assay for screening inpatients at high risk of MRSA	• No infection of interest
60	Lin et al. (2010)	Cost-effectiveness of influenza immunization in adult cancer patients in Taiwan	• Modeling<br>• No infection of interest
61	Lin et al. (2013)	Impact of an antimicrobial stewardship program with multidisciplinary cooperation in a community public teaching hospital in Taiwan	• No infection of interest
62	Lorente et al. (2011)	Lower associated costs using rifampicin-miconazoleimpregnated catheters compared with standard catheters	• No intervention of interest
63	Luan et al. (2011)	Universal prophylaxis is cost effective in cytomegalovirus serology-positive kidney transplant patients	• No infection of interest

64	Lusardi et al. (2013)	Antibiotic prophylaxis for short-term catheter bladder drainage in adults	• Review
65	Magalini et al. (2013)	Observational study on preoperative surgical field disinfection: povidone-iodine and chlorhexidine-alcohol	• No full economic evaluation
66	Matsushima et al. (2011)	Pre-emptive contact precautions for intubated patients reduced healthcare-associated meticillin-resistant Staphylococcus aureus transmission and infection in an intensive care unit	• No full economic evaluation<br>• No infection of interest
67	McLaws et al. (2012)	Zero risk for central line-associated bloodstream infection: are we there yet?	• No full economic evaluation
68	Meddings et al. (2012)	Effect of nonpayment for hospital-acquired, catheter-associated urinary tract infection: a statewide analysis	• No full economic evaluation
69	Minhas et al. (2011)	Risk factors for positive admission surveillance cultures for methicillin-resistant Staphylococcus aureus and vancomycin-resistant enterococci in a neurocritical care unit	• No full economic evaluation<br>• No infection of interest
70	Murthy et al. (2010)	Cost-effectiveness of universal MRSA screening on admission to surgery	• No infection of interest<br>• Modeling
71	Nelson et al. (2010)	Cost-effectiveness of adding decolonization to a surveillance strategy of screening and isolation for methicillin-resistant Staphylococcus aureus carriers	• No infection of interest
72	Nyman et al. (2011)	Cost of screening intensive care unit patients for methicillin-resistant Staphylococcus aureus in hospitals	• No infection of interest
73	Oncel et al. (2012)	Respiratory syncytial virus prophylaxis in preterm infants: a cost-effectiveness study from Turkey	• No infection of interest
74	O'Sullivan et al. (2009)	Cost-effectiveness of posaconazole versus fluconazole or itraconazole in the prevention of invasive fungal infections among neutropenic patients in the United States	• No infection of interest<br>• No intervention of interest
75	O'Sullivan et al. (2012)	Cost-effectiveness of posaconazole versus fluconazole for prevention of invasive fungal infections in US patients with graft-versus-host disease	• No infection of interest<br>• No intervention of interest
76	Ozgun et al. (2010)	Peri-operative antibiotic prophylaxis: adherence to guidelines and effects of educational intervention	• No full economic evaluation
77	Perez et al. (2013)	Integrating rapid pathogen identification and antimicrobial stewardship significantly decreases hospital costs	• No intervention of interest
78	Platt et al. (2010)	Cluster randomized trials in comparative effectiveness research: randomizing hospitals to test methods for prevention of healthcare-associated infections	• No full economic evaluation
79	Rijen et al. (2009)	Costs and benefits of the MRSA Search and Destroy policy in a Dutch hospital	• No infection of interest
80	Robotham et al. (2011)	Screening, isolation, and decolonisation strategies in the control of meticillin resistant Staphylococcus aureus in intensive care units: cost effectiveness evaluation	• No infection of interest
81	Samransamruajkit et al. (2010)	Effect of frequency of ventilator circuit changes (3 vs 7 days) on the rate of ventilator-associated pneumonia in PICU	• No intervention of interest
82	Scheetz et al. (2009)	Cost-effectiveness analysis of an antimicrobial stewardship team on bloodstream infections: a probabilistic analysis	• Modeling
83	Shorr et al. (2009)	Cost-effectiveness analysis of a silver-coated endotracheal tube to reduce the incidence of ventilator-associated pneumonia	• No intervention of interest
84	Simoens et al. (2009)	Search and destroy policy for methicillin-resistant Staphylococcus aureus: cost-benefit analysis	• No infection of interest
85	Slover et al. (2011)	Cost-effectiveness of a Staphylococcus aureus screening and decolonization program for high-risk orthopedic patients	• No infection of interest

86	Smith et al. (2010)	Cost-effectiveness of pneumococcal polysaccharide vaccine among healthcare workers during an influenza pandemic	• No infection of interest<br>• No intervention of interest<br>• Modeling
87	Stocker et al. (2012)	Antibiotic surveillance on a paediatric intensive care unit: easy attainable strategy at low costs and resources	• No intervention of interest
88	Stone et al. (2010)	Healthcare savings associated with reduced infection rates using antimicrobial suture wound closure for cerebrospinal fluid shunt procedures	• No infection of interest
89	Tubbicke et al. (2012)	Cost comparison of MRSA screening and management - a decision tree analysis	• Review
90	Vos et al. (2011)	Cost-effectiveness of routine (18)F-FDG PET/CT in high-risk patients with gram-positive bacteremia	• No intervention of interest<br>• No infection of interest
91	Wassenberg et al. (2011)	Cost-effectiveness of preoperative screening and eradication of Staphylococcus aureus carriage	• No intervention of interest
92	Yam et al. (2011)	Rethinking hospital general ward ventilation design using computational fluid dynamics	• No full economic evaluation
